# Learning Strategies Focused on Decision Making and Collaboration on Physical Education

**DOI:** 10.3390/ijerph17217924

**Published:** 2020-10-28

**Authors:** M.ª Alejandra Ávalos-Ramos, M.ª Ángeles Martínez Ruiz

**Affiliations:** Department of General and Specific Didactics, Faculty of Education, University of Alicante, 03690 Alicante, Spain; ma.martinez@ua.es

**Keywords:** university students, gymnastic skills, autonomy support, self-control

## Abstract

This research analyzes the voices of university students of sport sciences during the implementation of strategies to support autonomy and collaboration in gymnastic learning, from the perspectives of self-determination, self-control, and self-regulation. The methodology is qualitative and the self-reflective journals with their narrative are the tool to collect information. The strategy is well appreciated both in terms of the structure of the work plan and in the guidance of the tasks. The evolution of motivation, autonomy, collaboration, and achievements is highly valued throughout the process. However, the final assessment, despite having little effect on the grade, produces pressure and anxiety in students, so that self-control decreases, action is impaired, and the motivation achieved in the learning process is distorted. Further studies are needed to design coping strategies to help students maintain their motivation and confidence and to decrease students’ resistance to assessment tasks.

## 1. Introduction

### 1.1. Motivations and Resistances toward Physical Education in University Students

Within the framework of theories attempting to change behavior, specifically in the field of physical education (PE), the theory of self-determination has been especially researched [1]. We believe that the term determination is more accurate than motivation because it implies an important decision-making power. It is common for teachers to be able to get students motivated at first, but if they lack a strong driving force, the initial motivation may gradually diminish. There are many authors who develop and research these theories prolifically, Deci and Ryan [2], Ryan and Deci [1,3], as well as Reeve [4,5] in the field of motivation applied to teaching and learning, together with Haerens et al. [6] and Vansteenkiste et al. [7], among others. In general, all the authors mentioned identify three basic needs in students whose attention is a priority for teachers. The need for autonomy, the need for achievement and training, and the need for interrelationships.

The term autonomy, directly related to motivation, refers to the need for self-regulation of learning and has a social and practical meaning concerning the choice and involvement [8]. Secondly, Deci and Ryan [2] and Ryan and Deci [9] report that the need for training, competence, and achievement is a powerful incentive for an ongoing effort and perseverance. Therefore, it is better to get students to regulate themselves internally than to rely on reward and punishment. There are autonomy-oriented personalities who regulate their behavior in terms of possibilities and choices. Those who are control oriented, instead, regulate their conduct by focusing on rewards and punishments. It is thus very important in the teaching of PE to support autonomy and to avoid encouraging external motivation, which always makes individuals more dependent on the context [10]. As a third element, they establish the need for relationships, a powerful engine that pushes us to interact with others and feel connected to others. Curran et al. [11] analyzed the theory of SDT self-determination and found that when, for example, coaches used support for autonomy and interpersonal control in teams, greater involvement was more likely to follow. The need for enjoyment, closely linked to socialization, was also highlighted by several research studies, as a motivation to invest effort in a task. Woods et al. [12] found enjoyment to be the most influential motive affecting participation in physical activity and PE.

In short, the self-determination approach emphasizes that these needs help to establish commitment to tasks and must be considered and used in the learning of PE. We agree that these initial needs and motivations must be turned into stable capacities and they need, therefore, adequate learning strategies. For instance, autonomy is a must, but if it is not worked on and exercised, it loses effectiveness, and since its real implementation in different contexts and situations can lead to difficulties, some students may opt for dependency. Likewise, one can also take into consideration whether there are other related needs such as self-esteem or pleasure and the relationship between ability, motivation, and opportunity [13] as they are also determining factors.

Numerous studies, with greater or lesser connection to the self-determination theory framework, concur that positive attitudes were strongly associated with obtaining physical, personal, and social benefits from PE [14,15], which clearly corresponded to the students’ needs. Nevertheless, considering that benefits demand effort, a motivation must be defined by the conscious assessment of physical activity, which implies a cognitive and mental action [16] if we want to give rise to a continuous practice of physical and sport activity in the future.

### 1.2. Context of the Research: The Implementation of the Strategy to Support Autonomy and Collaboration in the Gymnastic Field

PE teachers find it difficult to move from the old pedagogies to the new ones, even though the latter allow them to anticipate students’ responses, know their needs, and encourage them in taking responsibility and making independent decisions, which leads them to report greater perceived enjoyment and competence compared to PE lessons delivered through traditional direct instruction [17]. New educational trends known as model-based practice or pedagogical models are gradually being implemented such as the Sports Education Model [18], the activist pedagogical model that aims to offer learning options and possibilities for all and challenge stereotypes [19] or student-centered research models as a curriculum that combines student and teacher actions, taking into account the voice of the students and the social construction of the content to be developed in the curriculum [20]. The teaching methodology by itself will not achieve greater effectiveness in student learning, that will also depend on the pedagogical skills and knowledge of the teacher and needs to be supported by a community of practice intent on improving learning across multiple domains in PE [21]. It is important that teachers generate self-reflection spaces and spaces where they can discuss and share experiences, challenges, and solutions on teaching–learning processes to encourage pedagogical change [22,23]. Evidence suggests that some teachers are trying to adopt a new model but are not succeeding in creating an appropriate learning environment. Consequently, the methodology cannot be seen as the only variable or as the most efficient one. Teachers often abandon new methodologies because they do not see the long-term benefits and they only want very short-term benefits [24]. On the contrary, teachers who work in a team find it easier to work on a new methodology, those who work in isolation tend to quit with the first difficulties. In any case, methodologies based on different models are now recommended, which allows a greater diversification in the functions of the teacher [25].

Collaborative work has often been used along with autonomy and decision-making strategies in the learning of gymnastic skills, as it enables the team to collaborate for a common purpose [26]. The team itself offers almost immediate feedback to its members and the contributions are interconnected and added up as has happened in other collaborative studies carried out in the context of physical activity and sport [26,27,28]. Likewise, in the gymnastic context, the collaborative work allows the reinforcement of the students’ skills, increasing their satisfaction as well as their predisposition toward the gymnastic activities [29,30], enabling all-round competency development of the students [28]. Sharing responsibilities and persevering in facing and solving difficulties will allow them to learn in a more authentic context.

Based on this conceptual framework, the aim of this study was to assess a teaching intervention to support autonomy and collaboration as a means of achieving motivation and satisfaction in gymnastic learning through the analysis of students’ reflective journals.

## 2. Materials and Methods

When we want to understand what the participants perceive, believe, feel, or experience, looking at meanings is the most appropriate, therefore the interpretive approach, usually called narrative or interpretative inquiry, is required [31]. PE research focus on themes such as motivation, resistance, perceived barriers, perceived competence, achievement expectations, or the level of self-regulation, which are examples of the various lines that can be investigated with interpretative approaches. The narrative inquiry approach allows the use of life stories as research data, since the personal narratives contain a force that give consistency to research [32].

This qualitative study had an intentional sample. We invited 38 students, aged 18–32 with a mean age of 19.92, SD = 2.869; from the 1st year of the bachelor’s degree in Physical Activity and Sport Sciences (PASS) of the Spanish University, who enrolled the subject of Gymnastic and Artistic Skills. The selected participants had to attend all the practical sessions of the subject. The narrative design of the research has made it possible to analyze, through the voices of university students, their experiences in the field of gymnastics in their own educational context [33], which will make it possible to identify possible actions and changes in university training practices.

### 2.1. Design of the Teaching Intervention

The methodological design consisted of the development of 13 practical learning sessions of the mentioned subject, in which a strategy of support to the autonomy in collaboration was implemented. Researchers and teaching staff of the subject designed and planned the basic structure of the proposal prior to the intervention. During the development of the intervention, consultations and discussions were held with the students about the activities to be carried out, always in a climate of listening and exchanging proposals. The organization was of 6 groups of 6–7 students each. All students played three roles: performer, assistant, observer–reporter. After each practice, students conducted and handed out a reflective journal of the session focusing on two aspects: relevant experiences and difficulties. The learning process was carried out in 3 phases (initial, progress, final) (Table 1):

In the initial phase, the first four sessions of the subject, initial assessment of basic gymnastic skills was carried out and progression exercises were performed. Similarly, main errors were identified as well as the protocols for the support of these elements. In this phase, information was provided on the methodological operations, i.e., creation of work teams, distribution of spaces, methodology to be used, and the function of the journals.

In the progress phase, consisting of six sessions, we worked on progressions of acrobatics of greater technical difficulty such as the roundoff or the backward roll to handstand. We developed the acrobatic gymnastics and, finally, we reviewed and clarified doubts. In addition, students in their groups worked on the competence to identify errors, on the feedback, and on the aids in a collaborative way.

In the final phase, last three sessions, main activities were evaluative. In this phase, final tests were prepared in groups according to the needs and in a mutually supportive way. The only test of individual technical execution of the subject was performed, which could have been prepared with the help of the group, and a test of resolution of practical assumptions was carried out in which each student had to play the role of assistant and observer.

### 2.2. Instrument

The instrument used for the data collection was a personal journal of each practice carried out. The personal journal as an instrument for qualitative research provides a wealth of information, allows a deep understanding of how people feel, and offers an important means of access to the rigorous study of educational processes [34].

Students were required to reflect after each session through a personal journal that revolved around the experiences of the session and the difficulties encountered during it. This journal was collected after each practice carried out. Students spent an average of 20 min completing their personal journal. Initially, 494 journals were collected. Finally, it was decided to only analyze 190 journals corresponding to sessions 1, 5, 6, 7, and 13 as they were the most representative of each phase, in the opinion of three experts in the subject under study.

Students were informed that the data from the personal journal could be used for research purposes. Informed consent was obtained, following the guidelines of the data protection law.

### 2.3. Procedure

The role of the researchers was to place themselves in the context of the participants, and to immerse in the observation and in the reading and interpretation of the class journal, to establish relationships between emerging keys of the narratives and conceptual framework that have guided the structure of the research. First, based on the selected journals, researchers made the first interpretative readings. Second, an initial categorization process was implemented that allowed the codes emerging from the students’ narratives to be connected to the conceptual framework and research questions [35]. Then, we moved on to the discussion and validation of the inferential codes through a process of triangulation to establish their reliability. In this stage, two specialists in PE and a university professor expert in gymnastics intervened, validating the codes and the definitive categories. Once these processes had been completed, after repetitive approaches, the final categories and codifications of this study were identified:

Category I. Gymnastics learning: motivations and dissatisfactions.

I.1 Motivations and evolution of satisfaction experiences in learning gymnastics.

I.2 Motivations and evolution of dissatisfactions in the learning of gymnastics.

### 2.4. Data Analysis

The data have been analyzed using software AQUAD 7 [36]. The possibilities of this program have allowed us to organize the categories and codifications extracted from the students’ reflections and thus to complement the qualitative analysis with a quantification. For this reason, we present, in the results, the code tables with the absolute frequency (AF) or number of findings regarding a concept and the percentage thereof (%AF).

## 3. Results

The results are organized according to the categories drawn from the research. In addition, some fragments of the students’ journals are shown, which exemplify the analysis codes.

### 3.1. Category I. Gymnastics Learning: Motivations and Dissatisfactions

PASS students mostly identify their learning experiences as satisfactory whereas there is less emergence of dissatisfaction during learning (Table 2). According to the sessions analyzed, we found the most positive perceptions in session six (76.6%) and seven (69.3%), and we identified the experiences with the most dissatisfaction in session thirteen (58.1%), where a test for the resolution of practical assumptions was carried out.

As a performer, I felt very good about myself, I have mastered the handstand exercise, as well as other movements such as the cartwheel with one hand. In the past I could not perform them, now I just need to perfect them. I have loved the class, the circuit, combining all kinds of movements (Student_10_S6).

After taking the exam, I was a little disappointed with myself, I failed some aids, this should have not happened (Student_37_S13).

**Figure 1:**
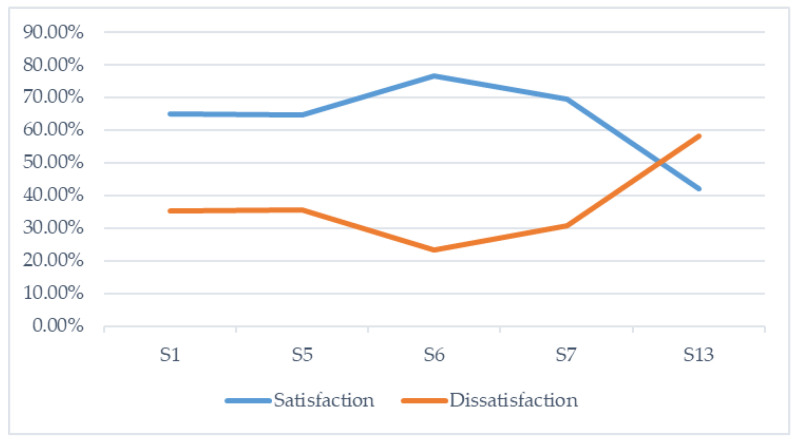
Evolution of the perceptions of satisfaction and dissatisfaction in the analyzed sessions of the teaching-learning gymnastic process.

As we observe in Figure 1, the satisfactions gradually increase until they reach the final test (Session 13) where the pressure of the assessment situation undoubtedly breaks their continuous and progressive motivation. Likewise, dissatisfactions decrease during the process. However, from Session 7 onward, the increasing oscillation of these dissatisfactions can be observed, possibly associated with the preparation of the acrobatic gymnastics test carried out in Session 8.

#### 3.1.1. Motivations and Evolution of Satisfaction Experiences in Gymnastics Learning

The satisfaction derived from the experiences (Table 3) is motivated by the gymnastic capacity that students perceive when they can perform the different activities proposed. This was particularly noticeable in sessions six (47%), seven (47.4%), and thirteen (46.7%). On the other hand, participants pointed out as a cause of satisfaction the working methodological proposal since it generates expectations of gymnastics learning, being more intense in session one (36.5%) and five (25.2%). Furthermore, the group’s peers are a source of satisfaction, which is reflected by the bonds and support created with the class group. These motivations are especially visible in session six (25.3%) and seven (23.1%). Another cause of satisfaction perceived by students is in the decision-making process during the gymnastic tasks proposed. This is an opportunity offered to students to be able to manage their own process by solving diverse learning situations associated with the detection of errors, the design and practical application of progression exercises, or the implementation of aids, as appropriate. In this sense, a greater presence of this cause is perceived in session thirteen (21.2%) and seven (15.3%). Finally, and with less presence, there are reasons for satisfaction related to the teaching staff, with a greater presence in session six (4.2%).

These satisfactions are reflected in the following excerpts from the students’ journals:

I performed the acrobatics quite well, I felt capable of improving in all those areas that helped to execute other exercises correctly. It is a very responsible role within this practice test as we had to perform the elements as best as possible (Student_09_S13).

I ended up feeling happy, I left with the feeling that this is going to be a subject in which I can learn many things and I am going to do some other things that I would never have imagined (Student_37_S1).

I’m very happy, since the creation of this piece involves integrating a series of values such as trust, companionship, empathy, listening... and at the same time you have the opportunity to meet your peers in another of their facets. I am very comfortable with my team and I think we are going to do a good job (Student_02_S7).

Then they made us do another series with aids and it worked out better, they left us some time to decide how we were going to do the aids. I was very happy with how I had solved the problem (Student_24_S13).

I would highlight the inclusion displayed by the acrobatic gymnastics and the cognitive aspect that it involves. We must make our own choreographies, considering all the details when choosing music, costumes, figures. The importance of adjusting the safety guidelines or the artistic phase, among others (Student_08_S7).

I would like to point out something that I like very much in all the sessions, which is the positive reinforcement offered by the teacher with feedback comments. I am aware of my limitations, but she always congratulates us when we do something well and values our progress however small. That is to be thanked (Student_29_S6).

**Figure 2:**
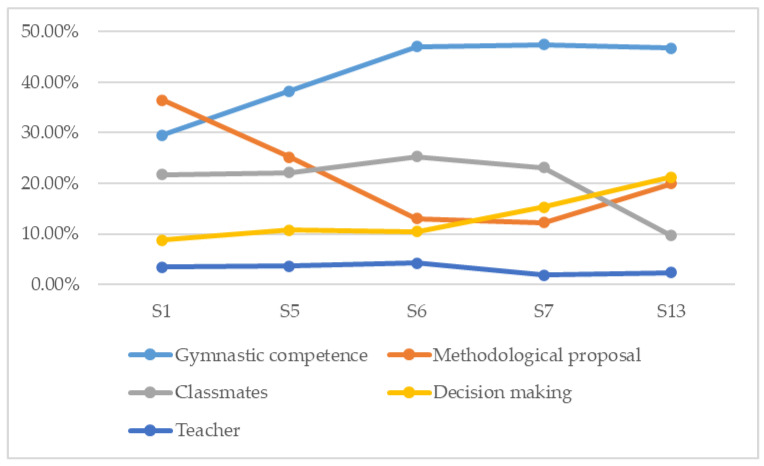
Satisfaction in gymnastics learning related to the perceptions about the learned gymnastic competence, the methodology proposal, the decision making, the classmates, and the teacher.

As we see in Figure 2, the reasons for satisfaction with a growing trend are the perceived improvement in their gymnastic competence, the bonds they create with their group classmates, and their ability to make decisions. On the other hand, we observe how the satisfaction with the methodological proposal decreases as the content develops and other achievements are accomplished, as we have mentioned. Teachers are not an indicator of satisfaction by themselves; their role is secondary due to the characteristics of the project in which the students are the main actors.

#### 3.1.2. Motivations and Evolution of Dissatisfactions in Gymnastics Learning

The causes of dissatisfaction indicated by the university students throughout the process of learning gymnastics (Table 4) were, among others, the deficiencies in the skills presented and the gymnastic incompetence that they felt especially in the fifth (59%) and sixth sessions (56.6%). Moreover, they have pointed out as negative causes, the lack of confidence, the fear, frustration, and perceived anxiety during the sessions held, especially in the initial (34.1%) and final sessions (59.9%). Group classmates are also mentioned with a negative perception. Lack of support or commitment from their peers has been reported, although with less presence. This perception was especially displayed in the seventh session (10.7%) and in the initial session (10.4%). To conclude, the muscular discomfort perceived especially in session five (7.9%) and seven (7.5%) together with the dissatisfaction with the teachers, exclusively in session thirteen (1.3%) are causes minimally mentioned. 

Below are excerpts from the students’ journals that reflect these dissatisfactions:

I keep improving the cartwheel, as I started from scratch and I have never been able to do it before. It’s very hard for me and I have difficulty in the roundoff. In the handstand, I lack more security in the gesture and some physical condition (Student_04_S5).

I find the roundoff complicated because of the position of the hands and the end with the corvette, since I end up doing the cartwheel or I don’t manage to raise my legs completely (Student_23_S6).

I felt a little afraid at first during the initial level test, due to the uncertainty of not knowing (Student_34_S1).

I got nervous during the exam and lacked responsiveness. Due to these nervousness, I have made very absurd mistakes that I don’t usually make, for example in the aid of the forward roll I have blocked myself and I haven’t been able to do it well until I managed to calm myself down (Student_26_S13).

At the end of the class we were given some time to get organized in the acrobatic gymnastics’ activity, where I could not execute any figure because most of my group was not there (Student_20_S7).

I have observed that my classmates had similar difficulties with the execution of the project, which did not help us at all, and that some of them lacked initiative in acting and helping (Student_10_S1).

Quite dissatisfied with the completion of the bridge as I can’t get it done. Also, it causes me a lot of pain in my lumbar area (Student_18_S7).

I think we should have been tested some other time during the term, to be able to deal with the insecurity and pressure that such a situation automatically causes (Student_02_S13).

It should be noted that there are student statements that specify no reason for dissatisfaction and that this was most noticeable in session six (18.6%) and in session seven (8.8%).

Today’s session went very smoothly and without any problems. At first, I was fine while we were practicing (Student_03_S6).

**Figure 3:**
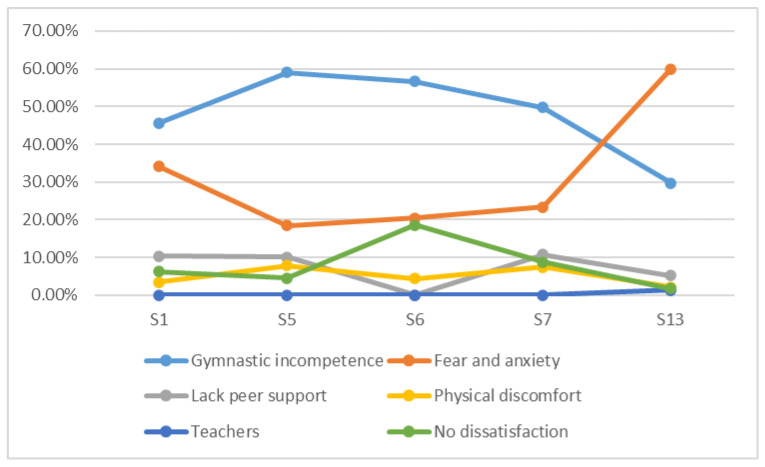
Dissatisfaction in gymnastics learning related to the perceptions about the gymnastic competence, fears and anxiety, lack of peer support, physical discomfort, teacher, and no dissatisfaction.

As we can see in Figure 3, at the beginning of the learning process, dissatisfaction grows due to the deficiencies and difficulties in performing gymnastic tasks. This perception begins to diminish after the fifth week of work and is less visible in the last weeks of training. On the other hand, the causes of dissatisfaction associated with anxiety, fears, or frustration decrease progressively until the exam preparation period begins, and they become exponentially accentuated in the final sessions where students were immersed in the evaluation process. 

We would like to stress that in the middle weeks participants reach their maximum in the non-perception of dissatisfaction, which decreased toward the end of the process.

## 4. Discussion

The appreciation of the methodological strategy is highly valued by the students when the teacher proposes it at the beginning, but during the process the references to the proposal decrease and, in their journals, they focus more on the specific variables. The lack of motivation with the methodological proposal is not present in the narratives either. These assessments are built from the opportunities offered by the group work allowing students to experience different roles in their learning. Not only is the performance of the acrobatic elements fundamental, but it is also important to highlight the observation of peers for the identification and correction of errors, in addition to offering direct assistance in the performance of the different tasks. We agree with O’Leary and Griggs [28] on the importance of sharing responsibilities and developing a social and emotional maturity allowing them to teach and learn from each other. Likewise, offering control situations in activities and training the self-control in high-pressure scenarios will guarantee less anxiety in students, especially when the evaluation is more latent and is presented as a challenge and not as a threat [37], just as it can happen in sports competitions where it is important to focus on the problem situation and seek support from others [38,39]. 

This can contribute to quality training not only on a technical level, but also to develop strategies for students to overcome obstacles and tools to cope with difficulties thanks to reflective and collaborative work, among other factors [40,41]. 

We can confirm that students reflect positively on the mastery and progression of their gymnastic skills since, in relation to the evolution of the specific variables of motivation, gymnastic competence grows from the initial session to the last. However, the highest percentage appears at the halfway point of the development of the strategy where there was no pressure on the task. In this regard, learning during teacher training from one’s own practical experience will be able to connect optimal teachings in one’s professional future [42]. On the other hand, the dissatisfaction with the gymnastic achievements grows during the first sessions due to the perception of difficulty in the proposed tasks, which can cause anxiety, stress, and insecurity in the movements, especially in the initial phase of the process. The feeling of being overwhelmed and other negative emotions diminish during the entire phase of progress until the end of their training, except for the last session where they emerge again. It is quite clear that the perceptions during the trajectory change [23] and the reasons for the change happen when the tasks become more difficult, and when they must prepare actions that require a performance. The emotional states that come with gymnastics learning must be considered since they are decisive for the technical acquisition and the cognitive stage of the learning process, especially in the first phases of gymnastics learning [43]. Several studies on frustration and dissatisfaction [44,45] have shown that the causes of dissatisfaction may lie in the lack of communication, lack of organization in the task, and lack of integration in the team, in addition to the initial lack of motivation as a result of the difficulties. In our analysis, we sought the above-mentioned elements.

The peer support variable progresses from the beginning to drop abruptly in the assessment session of the practice resolution where they preferred not to seek peer support. Their reflections are generally positive, although they refer more to issues of collaborative work than to expressions of enjoyment of a socially shared space. To some extent this diverges from the findings of Subramaniam and Silverman [46], who concluded that students’ enjoyment and their attitudes toward the practice would be enhanced if they were provided with a more suitable learning environment and added that in this way students are more likely to enjoy their experiences and maintain their intrinsic motivation. One would expect, according to many research studies that enhance the strength of socialization [39,47], that the need for relationships makes people highly motivated to be acknowledged. Therefore, between the control and support for autonomy, the support of the group predicts greater satisfaction in people because of their relational power. 

In the variable of the teacher support, there are not many allusions since the collaborative strategy carried out favors the autonomy, the interrelationship between students, and their direct involvement in the development of the gymnastic activities. Therefore, students have been autonomous, and the teaching role remains in the background, with their function being limited to observing, offering feedback, helping, and evaluating [48]. Their role is not so directive nor is it a protagonist in the learning scenario.

Autonomy and decision making are the only variable evolving upward during the entire process, although it is a rare category, probably because in the last evaluation session they necessarily had to make their own decisions. There is no statement expressly indicating resistance to autonomy or decision making. Although, is the appreciation of this opportunity was also not enhanced. This fact to some extent contradicts the perspectives that predict autonomy as a student need. We could dare to make some hypotheses to be confirmed in further studies. In this line, it is necessary to point out that the decision-making process of students in training is constantly changing as there are many factors that can influence this process such as the context or the cultural background of the students [23]. The Spanish university system grants considerable autonomy to the student by promoting the principles of European Convergence in the European Higher Education Space-EEA [49]. In primary education, even in secondary education, constructivist and innovative principles have been fully and deeply embraced in the methodological space of teaching and learning. This may mean that students, right from the start of their studies, are guaranteed a certain amount of autonomy. 

Even though there are numerous investigations in which the student shows his or her lack of motivation due to the inadequate methodology of the teaching staff [50], our experience in university teaching, and in research in the context of higher education, has shown us, on the contrary, that frequently our students of Physical Activity and Sport Sciences are quite flexible and sometimes react to the most innovative methods because they demand more effort than a conventional methodology. There are many students in first and second year of the bachelor’s degree who prefer more traditional methods as shown by the fact that master class format methodologies are rated positively in student satisfaction surveys. It is essential that teaching staff create spaces where they can share continuous actions and reflections with their students so that they can respond pedagogically to the needs and interests of a diverse student population [22,23].

In this case, the reflections in the class journal were maintained and did not diminish neither were they abandoned even in the moments of evaluation. This shows that communication and reflection strategies whether journals, self-talk [51] or think-aloud [52], and narrative interviews [53] are all working. Ekkekakis and Brand [54] insist that knowing how people feel when they are learning, and practicing is important to predict their perseverance. However, the “I feel better” effect is not always predictive, since emotional and affective responses are complex and past experiences can negatively influence on the perception of difficulty as can social, physical, and organizational or performance stressors [19,55]. Obviously, interpretive research requires the researcher to have dialogue, listening, and a reflective mood. Researcher must know and experience the context of the research to be able to interpret the information from a situated perspective. The interpretative perspective has been shown to be appropriate in this study in which researchers were also situated in the participants scenario. In short, seek deep meaning and make sense of experiences as Morse [56] stated; it is a promising innovation in PE.

Finally, we noted in the participants’ narratives that the strategy was useful and worth implementing. Our research has started from understanding that the implementation of an innovation strategy is to undertake a journey together with our students so that we can see their difficulties in each step, know them first-hand, and bring innovation in co-participation, in the same space and time. We can say that all researchers, teachers, and students have experienced innovation from within. We know about the gap that exists between research and applications, and we think that to bring together theory and action, we need a real immersion in the situation and in the path of innovation. However, within the limits of the study, the sample could be extended and variables that could influence the process such as the teaching staff or the gender of the students [19,24], as well as previous experience in this type of skills, could be analyzed. In our future lines of research, we plan to design, implement, and analyze methodological strategies that will allow us to manage the states of stress and anxiety of students at different times of the initial, continuous, or final assessment. Carrying out simulated test practices, role-playing that brings students closer to the type of test, and providing spaces for dialogue to share experiences of passing exams could be useful strategies to consider for future educational interventions.

## 5. Conclusions

From the beginning of the Gymnastic Skills subject, and after knowing the objectives and the learning process, students showed in their reflection’s good expectations and motivations, related to the learning strategy and the expected achievements. In the first and second phase, satisfaction grew while difficulties diminished. The experiences shared focused on the students’ satisfaction with respect to their competence and self-confidence, while controlling their fears and insecurities. The methodology was highly valued for offering them freedom, companionship, and competence, although some narratives reflected disagreements within the group and some difficulty. The problem, unexpected to a certain extent by the teachers, was the decrease in motivation and satisfaction due to not having enough skills to control the pressure of the final tests. Teachers should strive to create environments that are task-oriented, motivating, goal-oriented, and reflective in terms of self-perception of the ability to work under pressure, so that students will be more involved in the process of their learning, and their performance will be more effective.

In summary, we conclude that further research is needed on the above strategies to prevent learning under pressure from affecting and counteracting the results of an autonomous and collaborative learning process. We also hope that we have contributed modestly to the research on how to apply and implement perspectives to improve teaching and learning in a real and authentic context.

## Figures and Tables

**Table 1:** Contents developed in the sessions of the analyzed gymnastic skills.

Sessions	Main Activities
Session 1 (initial phase)	Explanation of how the subject works. Diagnostic evaluation of individual performance: forward roll, backward roll, cartwheel, and handstand. Group formation and decision making in the work group to practice gymnastic motor actions by pairs, quartets, and sextets.
Session 5 (progress phase)	Practice of acrobatic skills in groups: Group and individual decision making for design, implementation, and error detection and correction of diverse gymnastic tasks (connection and acrobatic elements). Practice of activities associated with progression exercises to learn the backward roll to handstand and the roundoff (decision making and implementation).
Session 6 (progress phase)	Practice of acrobatic skills in groups: Decision making for the design and implementation of circuits with six stations. Practice of progression exercise activities to learn vertical flip, the backward roll to handstand, and the roundoff (decision making and implementation). Group design of an acrobatic gymnastics practice (decision making and implementation).
Session 7 (progress phase)	Practice in groups. Decision making and implementation for the performance, assistance and error detection in the cartwheel, the handstand, the roundoff, the backward roll to handstand, and the vertical bridge. Practical design of acrobatic gymnastics prior to the group practical test of this discipline in session 8. Design decision making and implementation.
Session 13 (final phase)	Final test of resolution of 4 practical scenarios of the competencies developed in relation to the assistant and observer role. Individual and group qualification.

**Table 2:** Perception of gymnastics learning, according to sessions.

Codes	Session 1		Session 5		Session 6		Session 7		Session 13	
	AF	%AF	AF	%AF	AF	%AF	AF	%AF	AF	%AF
Satisfaction	318	64.8%	325	64.6%	370	76.6%	359	69.3%	165	41.9%
Dissatisfaction	173	35.2%	178	35.4%	113	23.4%	159	30.7%	229	58.1%
Total	491		503		483		518		394	

AF: absolute frequency.

**Table 3:** Motivations for satisfaction with gymnastics learning, by session.

Codes	Session 1		Session 5		Session 6		Session 7		Session 13	
	AF	%AF	AF	%AF	AF	%AF	AF	%AF	AF	%AF
Gymnastic competence	94	29.5%	124	38.2%	178	47%	170	47.4%	77	46.7%
Methodological proposal	116	36.5%	82	25.2%	49	13%	44	12.3%	33	20%
Classmates	69	21.7%	72	22.1%	96	25.3%	83	23.1%	16	9.7%
Decision making	28	8.8%	35	10.8%	40	10.5%	55	15.3%	35	21.2%
Teacher	11	3.5%	12	3.7%	16	4.2%	7	1.9%	4	2.4%
Total	318		325		379		359		165	

**Table 4:** Motivations for dissatisfaction in gymnastics learning, by session.

Codes	Session 1		Session 5		Session 6		Session 7		Session 13	
	AF	%AF	AF	%AF	AF	%AF	AF	%AF	AF	%AF
Gymnastic incompetence	79	45.7%	105	59%	64	56.6%	79	49.7%	68	29.7%
Fear and anxiety	59	34.1%	33	18.5%	23	20.4%	37	23.3%	137	59.9%
Lack peer support	18	10.4%	18	10.1%	0	0%	17	10.7%	12	5.2%
Physical discomfort	6	3.5%	14	7.9%	5	4.4%	12	7.5%	5	2.2%
Teachers	0	0%	0	0%	0	0%	0	0%	3	1.3%
No dissatisfaction	11	6.3%	8	4.5%	21	18.6%	14	8.8%	4	1.7%
Total	173		178		113		159		229	
